# The Prognostic Role of Baseline Eosinophils in HPV-Related Cancers: a Multi-institutional Analysis of Anal SCC and OPC Patients Treated with Radical CT-RT

**DOI:** 10.1007/s12029-022-00850-y

**Published:** 2022-08-01

**Authors:** Margherita Rimini, Pierfrancesco Franco, Federica Bertolini, De Bari Berardino, Zampino Maria giulia, Vegge Stefano, Kalliopi Andrikou, Francesca Arcadipane, Martina Napolitano, Lavajo Vieira Buno, Gerardi Marianna Alessandra, Francesco Olivero, Filippo Ferreri, Umberto Ricardi, Stefano Cascinu, Andrea Casadei-Gardini

**Affiliations:** 1 Department of Oncology and Hematology, Division of Oncology, University Hospital Modena, Modena, Italy; 2 Department of Oncology - Radiation Oncology, University of Turin School of Medicine, Via Genova 3, 10126 Turin, Italy; 3 Radiation Oncology, Centre Hospitalier Universitaire de Besançon, 25000 Besançon cedex, France; 4 Radiation Oncology, Réseau Hospitalier Neuchâtelois, CH-2300 La Chaux-de-Fonds, Switzerland; 5 Division of Radiation Oncology, European Institute of Oncology IRCCS, Milan, Italy; 6 Radiation Oncology Department, IRCCS Ospedale Policlinico San Martino, Genoa, Italy; 7 Oncologic Department, Istituto Scientifico Romagnolo per lo Studio e la Cura Dei Tumori, IRCCS, Meldola (Forlì), Italy; 8 Oncologic Department, IRCCS San Raffaele Scientific Institute Hospital, 20019 Milan, Italy

**Keywords:** Squamous cell anal cancer, HPV, Oropharyngeal cancer, Eosinophils, Prognostic factor, Chemoradiotherapy

## Abstract

**Background and Aim:**

Anal squamous cell carcinoma (SCC) and oropharyngeal cancer (OPC) are rare tumors associated with HPV infection. Bioumoral predictors of response to chemoradiation (CT-RT) are lacking in these settings. With the aim to find new biomarkers, we investigated the role of eosinophils in both HPV-positive anal SCC and HPV-related oropharyngeal cancer (OPC).

**Methods:**

We retrieved clinical and laboratory data of patients with HPV-positive anal SCC treated with CT-RT in 5 institutions, and patients with locally advanced OPC SCC treated with CT-RT in 2 institutions. We examined the association between baseline eosinophil count (the best cutoff has been evaluated by ROC curve analysis: 100 × 10^9/L) and disease-free survival (DFS). Unadjusted and adjusted hazard ratios by baseline characteristics were calculated using the Cox proportional hazards model.

**Results:**

Three hundred four patients with HPV-positive anal SCCs and 168 patients with OPCs (122 HPV-positive, 46 HPV-negative diseases) were analyzed. In anal SCC, low eosinophil count (< 100 × 10^9/L) correlates to a better DFS (HR = 0.59; *p* = 0.0392); likewise, in HPV-positive OPC, low eosinophil count correlates to a better DFS (HR = 0.50; *p* = 0.0428). In HPV-negative OPC, low eosinophil count confers worse DFS compared to high eosinophil count (HR = 3.53; *p* = 0.0098). After adjustment for age and sex, eosinophils were confirmed to be independent prognostic factors for DFS (HR = 4.55; *p* = 0.0139).

**Conclusion:**

Eosinophil count could be used as a prognostic factor in anal HPV-positive SCC. The worse prognosis showed in HPV-positive patients with high eosinophil count is likely to derive from an unfavorable interaction between the HPV-induced immunomodulation and eosinophils, which may hamper the curative effect of RT.

**Supplementary Information:**

The online version contains supplementary material available at 10.1007/s12029-022-00850-y.

## Introduction

Squamous cell carcinoma (SCC) of the anal canal is considered a rare tumor, which accounts for only 2–4% of all lower gastrointestinal malignancies [1]. Up to 80–90% of anal SCC is associated with high-risk human papillomavirus (HPV)–type infections, in particular HPV 16 genotype [2, 3]. Nowadays, radiotherapy (RT) in combination with 5-FU and mitomycin is the standard of treatment for localized disease, with a loco-regional recurrence (LRR) rate ranging from 20 to 40% and overall survival (OS) rates at 3–5 years ranging from 65 to 78% [4–8]. Clinical factors such as skin ulceration, nodal involvement, and male gender have been identified as important prognostic factors for local control and survival [5]. Laboratory indexes are not currently validated for predicting the prognosis and guiding clinical choices in this setting. In the last few years, research interest has grown on the interplay between cancer, inflammation, and immune system, and several bioumoral immune-based prognostic scores, such as lymphocyte count, neutrophil–lymphocyte ratio (NLR), and platelet-lymphocyte ratio (PLR), have been identified as predictors of survival, recurrence, and treatment response in cancer patients [9–11].

As an example, pretreatment systemic inflammatory index (SII) and NLR levels were significantly correlated with DFS and response prediction in patients with anal SCC treated with concurrent CT-RT [12–14].

Eosinophils have emerged as crucial components of the inflammatory process and development of cancer.

Even if the role of peripheral eosinophils is still controversial in cancer, it has been demonstrated that tumor-homing eosinophils secrete chemoattractants thus driving t-cells into the tumor [15]. Moreover, activated eosinophils induce macrophage polarization to promote tumor rejection by the host immune system [15]; finally, eosinophil cationic proteins have been highlighted to have cytotoxic activity not only against pathogens, but also against some kinds of malignant cells [16].

The prognostic role of baseline eosinophils has been assessed in a large number of tumor types. High eosinophil count at baseline was shown to convey a better prognosis in renal cell carcinoma, melanoma, colorectal, lung, cervical, hepatocellular carcinoma, and pancreatic cancer, while the data are more controversial for breast cancer and lymphoma [17–26]. Moreover, in clinical contexts outside oncology (e.g., infections by the respiratory syncytial virus, HBV virus, and SARS-CoV-2), high eosinophil count showed to be associated with better outcomes in terms of viral clearance and patient survival [27–29].

The prognostic role of peripheral blood eosinophil count in anal SCCs receiving RT-CT remains to be investigated.

HPV has a central role in the pathogenesis of other types of cancer, including head and neck cancers (HNCs) and particularly oropharyngeal squamous cell carcinoma (OPC) [30, 31]. The HPV-positive subset of OPC is characterized by increased sensitivity to chemoradiotherapy protocols with a higher likelihood for response, and an overall better prognosis [32].

We hypothesized that the HPV virus infection may influence the blood eosinophil count and, consequently, its significance. In the composite interplay between cancer and host immune reaction, viral infection represents a further element of complexity which influences the panorama depending on its intrinsic characteristics. HPV, unlike other kinds of viruses involved in cancer development, has been highlighted to have immune modulation properties which could completely change the cancer microenvironment and, consequently, the role of the host immune cells.

With the aim to verify our considerations, we investigated the impact of eosinophil count on survival outcomes in patients affected with HPV-positive anal SCC, and in both HPV-positive and HPV-negative OPCs.

## Material and Methods

### Patient Selection: Anal Cancer Patients

We retrieved clinical data regarding patients treated for anal cancer at the Radiation and Medical Oncology Departments of 5 institutions: University of Turin, AOU Citta’ della Salute e della Scienza in Turin, Department of Modena Cancer Center, Università di Modena e Reggio Emilia, Ospedale San Martino Genova, Centre Hospitalier Régional Universitaire Jean Minjoz, Besancon, and European Institute of Oncology in Milan. Briefly, all patients had a histologically confirmed diagnosis of HPV-positive SCC located either within the anal canal or margin. The tumor stage was defined following the indications of the American Joint Committee on Cancer (2002 version), and patients with clinical stages T1-T4, N0-N3, and M0 were included. Patients having clinical T1N0 tumors of the anal margin were excluded, because they were treated with local excision. Patients were treated with concomitant chemoradiotherapy. Concomitant CT consisted of 5-fluorouracil (5-FU) (1000 mg/m^2^/day) given as continuous infusion for 96 h (days 1–5 and 29–33) combined with mitomycin C (MMC) (10 mg/m^2^) given as bolus (days 1 and 29). Mitomycin C was capped at 20 mg maximum. Alternatively, patients were treated with CT consisting of 5-FU 200 mg/m^2^ given as continuous infusion for 24 h combined with Cisplatin 80 mg/m^2^ (days 1 and 21). Radiotherapy was delivered using static or volumetric intensity-modulated approaches up to a total prescription dose to the macroscopic disease ranging between 50.4 and 59.4 Gy, depending on tumor size and stage. Elective nodal irradiation was offered to patients on pelvic lymph nodes and inguinal groins up to a conventionally fractionated dose of 45 Gy [12, 33].

### Patient Selection: Head and Neck Patients

We retrieved clinical data regarding patients treated for OPC at the Radiation and Medical Oncology Department of 2 institutions: University of Turin, AOU Citta’ della Salute e della Scienza in Turin, Department of Modena Cancer Center, Università di Modena e Reggio Emilia. Patients with locally advanced disease OPC were included in this study (stages III and IVA). In this study, the tumor stage was defined by the criteria used in the 7th edition TNM staging system. All patients had a histologically confirmed diagnosis of SCC and they were treated with concomitant chemoradiotherapy (CRT) with single-agent platinum with curative intent. Patients received cisplatin 100 mg/m^2^ on days 1, 22, and 43 or weekly cisplatin 40 mg/m^2^ over 6–7 weeks. Radiation was delivered with static or volumetric intensity-modulated radiotherapy. The most common RT schedule for definitive treatments was 70 Gy/35 fractions (2 Gy daily) to the macroscopic disease, 63 Gy/35 fractions (1.8 Gy daily) to the “intermediate-risk prophylactic volume,” and 54.25 Gy/35 fractions (1.55 Gy daily) to the “low-risk volume.”

Pretherapy tumor biopsies were assessed for high-risk HPV and HPV positivity was defined as p16 immunohistochemistry positive staining.

### Statistical Analysis

We investigated the correlation between baseline eosinophil count and disease-free survival (DFS) in patients affected with anal SCC and OPC.

Eosinophil count as well as all other laboratory exams were retrieved from the medical records provided, and they were performed within 1 week before treatment started. X-tile 3.6.1 software (Yale University, New Haven, CT) was used to determine the cutoff value for baseline levels. Relying on the results of the ROC curve, a cutoff point of eosinophil > 100 × 10^9/L was considered an elevated level.

Categorical variables were compared with Fisher’s exact test.

For anal SCC patients, DFS was defined as the time from the first day of CRT until clinical or radiologic disease recurrence, or death by any cause, or last follow-up visit. For OPC patients, DFS was defined as the time from the first day of CRT until clinical or radiologic disease recurrence, or death for any cause, or last follow-up visit.

DFS was estimated by the Kaplan–Meier method and curves were compared by the log-rank test. Unadjusted and adjusted hazard ratios (HRs) by baseline characteristics were calculated using the Cox proportional hazards model. MedCalc package (MedCalc® version 16.8.4) was used for statistical analysis.

## Results

Three hundred four consecutive patients with HPV-positive anal SCC treated from May 2007 to May 2018 were available for the analysis. Most of the patients were female (75.0%) with a mean age of 65 years. The most represented single global tumor stage was stage III (46.7%). The main characteristics of the patients enrolled in the study are summarized in Table 1.

**Table 1:** Patient characteristics

	Variable	*N* (%)
Age	Mean	64
	Range	33–93
Gender	Male	49 (16.1)
	Female	255 (83.9)
T-stage	T1	27 (9.0)
	T2	159 (52.4)
	T3	81 (26.7)
	T4	36 (11.9)
	NA	1 (< 0.1)
N-stage	N0	148 (48.7)
	N1	59 (19.5)
	N2	57 (18.9)
	N3	39 (12.9)
	NA	1 (< 0.1)
Global stage	I	16 (5.4)
	II	125 (41.2)
	III	162 (53.4)
	NA	1 (< 0.1)
Grade	G1	41 (13.5)
	G2	120 (39.5)
	G3	79 (26.0)
	NA	64 (21.0)
Eosinophil count	> 100 × 10^9/L	209
	< 100 × 10^9/L	97

One hundred sixty-eight patients with OPC treated from March 2007 to October 2018 were available for the analysis; of them, 122 patients (72.6%) were HPV-positive and 46 patients (27.4%) were HPV-negative. Amongst HPV-positive cases, most patients were male (70.7%) with a mean age of 63 years. Amongst HPV-negative cases, most of the patients were male (76.1%) with a mean age of 60 years. The main patients’ characteristics according to HPV status are summarized in Table 2.

**Table 2:** Patient characteristics in head and neck cancer cohort

	HPV-negative *N* (%)	HPV-positive *N* (%)	*p*-value
Gender			
Female Male	11 (23.9) 35 (76.1)	36 (29.3) 87 (71.7)	0.56
Age			
< 70 ≥ 70	39 (84.8) 7 (15.2)	93 (75.6) 30 (24.4)	0.21
Smoking			
Yes No NA	38 (82.6) 6 (13.0) 2 (4.4)	69 (56.1) 40 (32.5) 14 (11.4)	0.0059
Grading			
1–2 3 No data	18 (39.1) 17 (37.0) 11 (23.9)	20 (16.3) 66 (53.6) 37 (30.1)	0.004
Stage*			
II–III IVA NA	7 (15.2) 37 (80.4) 2 (4.4)	54 (43.9) 67 (54.5) 2 (1.6)	0.0004
Eosinophil			
< 100 × 10^9/L > 100 × 10^9/L	10 (21.7) 36 (78.3)	51 (41.5) 72 (58.5)	0.01

^*^TNM e’ 7th edition; *NA* not available

### Anal Cancer Patients

**Fig. 1:**
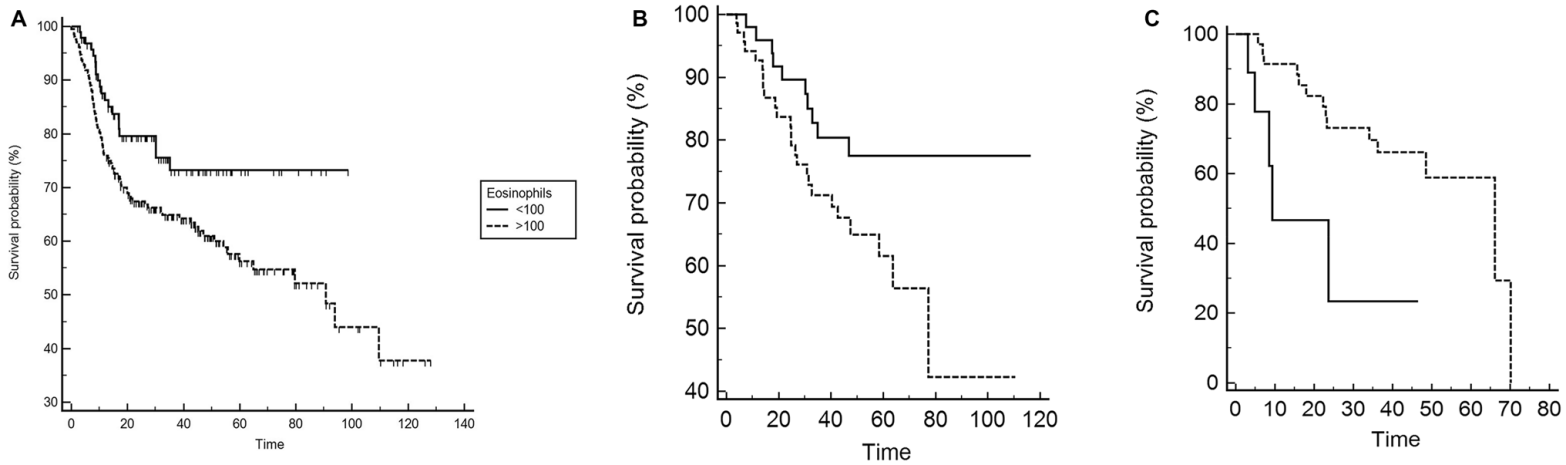
Kaplan–Meier curves for DFS in the anal cancer cohort (**A**), HPV-positive head and neck cancer cohort (**B**), HPV-negative head and neck cancer cohort (**C**)

At univariate analysis, patients with low eosinophil count (< 100 × 10^9/L) compared to those with high baseline eosinophil count (> 100 × 10^9/L) had a better DFS (HR = 0.60; 95%CI: 0.40–0.93; *p* = 0.0219) (Table 3) (Fig. 1A).

**Table 3:** Univariate and multivariate analysis of DFS in the anal cancer cohort

	Univariate		Multivariate
	HR (95%CI)	*p*	
Boost (yes vs no)	0.93 (0.62–1.38)	0.73	
Age (< 70 vs > 70)	1.24 (0.82–1.88)	0.2970	
Grading (3 vs < 3)	1.10 (0.69–1.74)	0.6811	
Hemoglobin (< 12 vs > 12 gr/dl)	2.28 (1.32–3.93)	**0.0029**	0.0630
Node metastasis (yes vs no)	1.44 (0.98–2.11)	0.0626	
SII (< 560 vs > 560)	0.59 (0.40–0.87)	**0.0084**	0.0793
Stage (3 vs < 3)	1.55 (1.05–2.28)	**0.0246**	
T (4 vs < 4)	2.47 (1.32–4.62)	**0.0045**	0.0837
Eosinophil (< 100 vs > 100 × 10^9/L)	0.60 (0.40–0.93)	**0.0219**	**0.0392**

Bold entry indicates the statistical significance

The other parameters associated with DFS were as follows: patients with hemoglobin < 12 gr/dl versus those with hemoglobin > 12 gr/dl and patients with T stage 4 versus those with T2-T3 stage had a worse DFS (HR = 2.28; 95%CI: 1.32–3.93; *p* = 0.0029; HR = 2.47; 95%CI: 1.32–4.62; *p* = 0.0045; respectively); conversely, patients with systemic inflammatory index < 560 versus patients with systemic inflammatory index ≥ 560 had a better DFS (HR = 0.59; 95%CI: 0.40–0.87; *p* = 0.0084) (Table 3).

Following adjustment for clinical covariates positive at univariate analysis, multivariate analysis confirmed eosinophils as an independent prognostic factor for better DFS (HR = 0.59; 95%CI: 0.36–0.97; *p* = 0.0392) (Table 3).

Differences were found between eosinophil count < 100 × 10^9/L and > 100 × 10^9/L also in terms of complete response (85.6% vs. 73.5%; odds ratio 0.46; 95%CI 0.24–0.88; *p* = 0.02).

Moreover, blood eosinophil count was significantly correlated with lymphocytes (*p* = 0.001) (Supp. Figure 1A) and no correlation with neutrophils was found (*p* = 0.287) (Supp Fig. 1B).

### HPV-Positive OPC Patients

At univariate analysis, patients with low baseline eosinophil count (< 100 × 10^9/L) had a better DFS with a HR = 0.50 (95%CI: 0.25–0.99; *p* = 0.0428) compared to patients with high eosinophil (> 100 × 10^9/L) (Table 4) (Fig. 1B).

**Table 4:** Univariate and multivariate analysis of DFS in the head and neck cancer cohort

	HPV-positive			HPV-negative		
	Univariate analysis		Multivariate	Univariate analysis		Multivariate
	HR (95%CI)	*p*	*p*	HR (95%CI)	*p*	*p*
Sex (female vs male)	1.05 (0.51–2.17)	0.8797	0.4672	0.87 (0.32–2.37)	0.8058	0.5545
Age (< 70 vs > 70)	0.84 (0.38–1.86)	0.6758	0.9215	0.69 (0.16–2.83)	0.5532	0.8570
Habit smoking (yes vs no)	2.72 (1.23–5.99)	**0.0127**	**0.0293**	1.22 (0.31–4.78)	0.7831	
Grading (3 vs < 3)	0.98 (0.35–2.67)	0.9690		1.19 (0.44–3.21)	0.7152	
N (N0 vs N +)	0.96 (0.23–4.12)	0.9556		0.46 (0.15–1.43)	0.2964	
Eosinophil (< 100 vs > 100 × 10^9/L)	0.50 (0.25–0.97)	**0.0428**	0.4198	3.52 (0.74–17.71)	**0.0098**	**0.0139**

Bold entry indicates the statistical significance

The other parameters associated with DFS were as follows: patients without smoking habits versus those with smoking habits had a better DFS (0.37; 95 CI 0.17–0.81; 0.0127); conversely, an increase of neutrophils had a worse DFS (1.20; 95%CI 1.00–1.44) (Table 4).

Following adjustment for clinical covariates positive in univariate and multivariate analysis, there were no confirmed eosinophils as independent prognostic factors for DFS (HR = 1.39; 95%CI: 0.62–3.10; *p* = 0.4198) but the habit of smoking was the only positive variable (HR = 3.5; 95%CI: 1.13–10.80; *p* = 0.0293) (Table 4).

Moreover, blood eosinophil count had a correlation trend with lymphocytes (*p* = 0.086) and no correlation with neutrophils was found (*p* = 0.781).

### HPV-Negative OPC Patients

At univariate analysis, patients with low baseline eosinophil count (< 100 × 10^9/L) had a worse DFS with a HR = 3.53 (95%CI: 1.97–16.71; *p* = 0.0098) compared to patients with high eosinophil (> 100 × 10^9/L). No other parameters were associated with disease-free survival (Table 4) (Fig. 1C).

Following adjustment for age and gender, multivariate analysis confirmed eosinophils as independent prognostic factors for DFS (HR = 4.55; 95%CI: 1.36–15.22; *p* = 0.0139) (Table 4).

The blood eosinophil count was significantly correlated with lymphocytes (*p* = 0.012) and no correlation with neutrophils was found (*p* = 0.639).

**Fig. 2:**
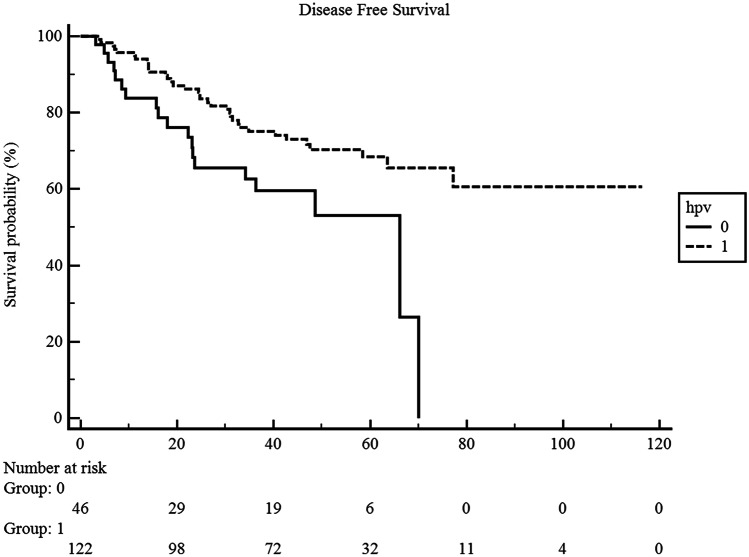
Kaplan–Meier curves for DFS according to the HPV status (HPV-positive vs HPV-negative in OPC cohort of patients)

Finally, we compared the DFS in patients with HPV + and HPV- OPC, thus showing a significantly better survival outcome in patients with HPV positivity treated with radical CT-RT (Fig. 2).

## Discussion

In this study, we investigated the role of baseline blood eosinophil count in HPV-positive anal SCCs treated with chemoradiotherapy, and then in both HPV-positive and HPV-negative OPCs treated with platinum-based concurrent CT-RT.

We highlighted a positive prognostic impact of low baseline blood eosinophil count in both anal SCCs and HPV-positive OPCs; conversely, in HPV-negative OPC patients, low eosinophil count resulted to have a negative prognostic impact.

It could be hypothesized that the complex interplay between the HPV infection, the immune modulation effect conveyed by eosinophils, and the RT on the microenvironment and immune system could be almost related to the results we found. Human papillomavirus (HPV) is a double-stranded DNA virus involved in chronic inflammation, oxidative stress, and, consequently, carcinogenic process [34].

With the goal of evading immune recognition, HPV developed different strategies to establish an immune microenvironment characterized by a weak immune-responsive/strong immune-modulatory phenotype. The most important strategy of virus immune evasion is related to two oncoproteins E6 and E7, since recent evidence demonstrates that both these proteins can regulate transcription from the host genome of infected cells [35–39], in particular the innate immune genes [38–43] by the repression of NF-kB signaling. According to the downregulation of the NB-kB pathway, in HPV-positive tissues, it has been demonstrated a downregulation of proinflammatory cytokines, such as IL-1, IL-2, and IFN-ℽ, and an upregulation of immunosuppressive cytokines, such as IL-6, IL-10, and TGF-β [44–48]. Furthermore, E6 and E7 proteins were shown to selectively downregulate surface major histocompatibility complex (MHC) class I expression, and the Toll-like receptor 9 (TLR9) [49, 50], which are normally involved in binding exogenous viral DNA and triggering the proinflammatory cascade [51]. Eosinophils have been reported to have multiple roles as effectors and coordinators in both innate and adaptative immune responses. Indeed, they act as an effector by exerting a Th2 immune-depending proinflammatory action against pathogen infections or tissue injury, eventually due to tumors, but also regulate and interact with several immune cell population thus including T cells and dendritic cells. In the case of HPV-related tumors, eosinophils seem to act in synergy with the HPV infection, since they both contribute to creating a microenvironment with an immune-modulated phenotype by the elaboration of a Th2-type response and by the production of mediators including TGF-β1, MMPs, and proangiogenic factors (ex: VEGF), all involved in immunosuppression, carcinogenesis, and cancer progression [52, 53].

Marks et al. [54] described that, as a response to HPV infection, the host’s immunoregulatory cytokine seems to shift from IL-2 to eotaxin (subfamily of eosinophil chemotactic proteins), with a likely enhanced role of innate responses and a loss of regulation of antigen-specific adaptative responses, and the consequent eosinophil recruitment and activation in the local microenvironment. Surprisingly, in our study, the absolute total amount of peripheral eosinophils was observed to be higher in HPV-negative patients compared to HPV-positive cancers, highlighting the importance of the relationship between local and peripheral immune cells in cancer patients. Pieces of evidence in the literature seem to show a complex and not completely understood the correlation between markers of systemic inflammation and local immune infiltration in cancers [55, 56], but there are no current pieces of evidence that the peripheral immune cell count, including eosinophil count, could be a surrogate for the count of the same immune cells in the tumor microenvironment.

Given the eosinophil defense properties and the literature available on eosinophils and viral infections, it might be speculated that a low eosinophil count in HPV-positive cancer patients could be the expression of an ineffective immune response to the infection, which is likely to encourage the mechanisms of HPV-related cancer progression; alternatively, a low eosinophil count could be the expression of a high HPV viral load which lead to eosinophil consumption and, consequently, a worse prognosis.

Furthermore, an important consideration has to be done concerning the influences of different chemotherapy regimens in different settings. Indeed, patients analyzed in this study have been treated with different cytotoxic drugs, which present different hematologic toxicities (for example, mitomycin has been demonstrated to lead to myelotoxicity more frequently compared to the cisplatin-based regimen) and pharmacokinetic, even if both the settings considered see the combination of radiotherapy and chemotherapy as the backbone of therapy. For this reason, different influences on the immune system as well as on the tumor microenvironment have to be considered as a consequence of different regimens used.

Nevertheless, in our dataset, the immunological landscape is made even more complex by the role of radiotherapy on the immune system.

Radiotherapy is a mainstay local therapy in both anal SCC and OPC.

RT induces an “immunogenic cell death” (ICD), which causes the release of endogenous damage-associated molecular patterns (DAMPs). The DAMPs, which include calreticulin, high-mobility group box 1 protein (HMGB1), and adenosine triphosphate (ATP), contribute to the priming of the immune system by activating dendritic cells, improving antigen presentation to T cells [57, 58], and stimulating the production of proinflammatory cytokines TNF, IL-1, IL-6, and IL-8 [59]. Also, eosinophils are involved in the first response to the DAMPs, since they express on their surface the DAMPs receptors [60]. These mechanisms convert toward the creation of a strong inflammation process which contribute to the anti-tumoral effect of the RT. HPV-positive cancers are characterized by a high radio-sensitivity [61, 62], which seems to be correlated to an altered DNA repair system induced by the oncoproteins E6 and E7, reduced hypoxic regions which could contribute to the DNA indirect damage by free radicals [63–66] and increased cellular immune response which mediates an effective immune response following treatment [67, 68]. On the other hand, in HPV-positive cancers, the RT effect has to interface the immunomodulatory action of the virus on the cancer microenvironment, and the systemic immunological status.

In fact, if the mechanisms described constitute the foundation of radio-sensitivity of HPV-positive cancers, we have to note that the equilibrium between pro- and anti-inflammatory cytokines and the microenvironment status just prior to RT is critical, since it may influence the cancer resistance to the treatment. The anti-inflammatory action induced by eosinophils can weaken the RT-derived inflammation, thus resulting in a predominance effect of the HPV immunomodulation and in a weaker RT anti-tumoral effect leading to a worse prognosis. In favor of our hypothesis, Zhu et al. [69] reported a similar eosinophil-negative prognostic role in another HPV-related cancer setting: cervical squamous cell carcinoma. They analyzed a cohort of cervical squamous cell carcinoma patients treated with hysterectomy plus pelvic lymph node dissection and external irradiation, and concluded that higher pretreatment eosinophil count was independently correlated with worse PFS and OS (69).

In contrast, in HPV-negative cancers, where the immunomodulatory action induced by the virus lacks, a high eosinophil count contributes to an adequate inflammatory response to RT, and therefore to the anti-tumoral effect.

This study presents some limits: first of all, we reported data on the eosinophil count in peripheral blood, while data about the eosinophil count in the cancer microenvironment are lacking. Secondly, we considered the eosinophil count just prior to CT-RT treatment, but we did not report on the eosinophil count after treatment; thus, we do not get information about how eosinophil count changes during treatment and in response to RT in both HPV-positive and negative cancers. Finally, no data have been reported regarding the continuity in treatments, which means that a kind of bias could derive from the lack of information about patients who have eventually stopped treatments due to hematologic toxicities, and results have to be interpreted with quite attention. New studies are needed in order to deepen the dynamic role covered by eosinophils in the immune microenvironment of HPV-positive SCCs.

## Conclusion

In the complex interplay between HPV, immune microenvironment, and RT, the presence of high eosinophil count in HPV-positive cancers, both anal SCCs and OPCs, is likely to create an imbalance toward an anti-inflammatory microenvironment which frustrates the RT benefic effect, while the presence of low eosinophil count gives the go-ahead to RT to exert its proinflammatory effects and to counter the immune modulation derived by the virus. Our results highlight a possible use of the peripheral eosinophil count as a predictor of response to CT-RT in the anal SCC HPV-positive cancers setting.

## Supplementary Information

Below is the link to the electronic supplementary material.

Supplementary file1 (PPTX 1.52 MB)

## References

[1] Jemal A, Siegel R, Ward E (2008). Cancer statistics. CA Cancer J Clin.

[2] Daling JR, Madeleine MM, Johnson LG (2004). Human papillomavirus, smoking, and sexual practices in the etiology of anal cancer. Cancer.

[3] Frisch M, Glimelius B, van Den Brule AJ (1997). Sexually transmitted infection as a cause of anal cancer. N Engl J Med.

[4] UKCCCR Anal cancer Trial Working Party (1996). UK Co-ordination Committee on Cancer Research: Epidermoid anal cancer: results from the UKCCCR randomized trial of radiotherapy alone versus radiotherapy, 5-fluorouracil, and mitomycin. Lancet.

[5] Bartelink H, Roelofsen F, Eschwege F (1997). Concomitant radiotherapy and chemotherapy is superior to radiotherapy alone in the treatment of locally advanced anal cancer: results of a phase III randomized trial of the European Organization for Research and Treatment of Cancer radiotherapy and gastrointestinal cooperative group. J Clin Oncol.

[6] Flam M, John M, Pajak TF (1996). Role of mytomicin in combination with fluorouracil and radiotherapy, and of salvage chemoradiation in the definitive nonsurgical treatment of epidermoid carcinoma of the anal canal: results of a phase III randomized intergroup study. J Clin Oncol.

[7] Ajani JA, Winter KA, Gunderson LL (2008). Fluorouracil, mitomycin, and radiotherapy vs fluorouracil, cisplatin, and radiotherapy for carcinoma of the anal canal: a randomized controlled trial. JAMA.

[8] James RD, Glynne-Jones R, Meadows HM (2013). Mitomycin or cisplatin chemoradiation with or without maintenance chemotherapy for treatment of squamous-cell carcinoma of the anus (ACTII): a randomised, phase 3, open-label, 2 x 2 factorial trial. Lancet Oncol.

[9] Casadei Gardini A, Scarpi E, Faloppi L (2016). Immune inflammation indicators and implication for immune modulation strategies in advanced hepatocellular carcinoma patients receiving sorafenib. Oncotarget.

[10] Bruix J, Cheng A-L, Meinhardt G (2017). Prognostic factors and predictors of sorafenib benefit in patients with hepatocellular carcinoma: analysis of two phase III studies. J Hepatol.

[11] Casadei-Gardini A, Scarpi E, Ulivi P (2020). Prognostic role of a new inflammatory index with neutrophil-to-lymphocyte ratio and lactate dehydrogenase (CII: Colon Inflammatory Index) in patients with metastatic colorectal cancer: results from the randomized Italian Trial in Advanced Colorectal Cancer (ITACa) study [Corrigendum]. Cancer Manag Res.

[12] Casadei-Gardini A, Montagnani F, Casadei C (2019). Immune inflammation indicators in anal cancer patients treated with concurrent chemoradiation: training and validation cohort with online calculator (ARC: Anal Cancer Response Classifier). Cancer management and research.

[13] De Felice F, Rubini FL, Romano L (2019). Prognostic significance of inflammatory-related parameters in patients with anal canal cancer. Int J Colorectal Dis.

[14] Schernberg A, Escande A, Rivin Del Campo E (2017). Leukocytosis and neutrophilia predicts outcome in anal cancer. Radiother Oncol.

[15] Carretero R, Sektioglu IM, Garbi N (2015). Eosinophils orchestrate cancer rejection by normalizing tumor vessels and enhancing infiltration of CD8(+) T cells. Nat Immunol.

[16] Wong D, Winter O, Hartig C (2014). Eosinophils and megakaryocytes support the early growth of murine MOPC315 myeloma cells in their bone marrow niches. PLoS ONE.

[17] Zahoor H, Barata PC, Jia X, et al. Patterns, predictors and subsequent outcomes of disease progression in metastatic renal cell carcinoma patients treated with nivolumab. J Immunother Cancer. 2018;6(1):107. 10.1186/s40425-018-0425-8 PMC6192175 30333065

[18] Rosner S, Kwong E, Shoushtari AN (2018). Peripheral blood clinical laboratory variables associated with outcomes following combination nivolumab and ipilimumab immunotherapy in melanoma. Cancer Med.

[19] Wei Y, Zhang X, Wang G (2018). The impacts of pretreatment circulating eosinophils and basophils on prognosis of stage I-III colorectal cancer. Asia Pac J Clin Oncol.

[20] Tanizaki J, Haratani K, Hayashi H (2018). Peripheral blood biomarkers associated with clinical outcome in non-small cell lung cancer patients treated with nivolumab. J Thorac Oncol.

[21] Holub K, Biete A (2019). Impact of systemic inflammation biomarkers on the survival outcomes of cervical cancer patients. Clin Transl Oncol.

[22] Holub K, Conill C (2019). Unveiling the mechanisms of immune evasion in pancreatic cancer: may it be a systemic inflammation responsible for dismal survival?. Clin Transl Oncol.

[23] Ownby HE, Roi LD, Isenberg RR, Brennan MJ (1983). Peripheral lymphocyte and eosinophil counts as indicators of prognosis in primary breast cancer. Cancer.

[24] Utsunomiya A, Ishida T, Inagaki A (2007). Clinical significance of a blood eosinophilia in adult T-cell leukemia/lymphoma: a blood eosinophilia is a significant unfavorable prognostic factor. Leuk Res.

[25] Hude I, Sasse S, Bröckelmann PJ (2018). Leucocyte and eosinophil counts predict progression-free survival in relapsed or refractory classical Hodgkin lymphoma patients treated with PD1 inhibition. Br J Haematol.

[26] Orsi G, Tovoli F, Marisi G, et al. Prognostic role of blood eosinophil count in sorafenib-treated hepatocellular carcinoma patients: time to reconsider the minorities. Liver Cancer Summit 2020 (EASL). Article in press.

[27] Phipps S, Lam CE, Mahalingam S (2007). Eosinophils contribute to innate antiviral immunity and promote clearance of respiratory syncytial virus. Blood.

[28] Li Y, Zhao Y, Liu J (2010). A promising alternative anti-HBV agent: the targeted ribonuclease. Int J Mol Med.

[29] Yingzhen Du, Lei Tu, Pingjun Zhu, et al. Clinical features of 85 fatal cases of COVID-19 from Wuhan: a retrospective observational study. AJRCCM Articles in Press. Published April 03, 2020 as 10.1164/rccm.202003-0543OC Copyright © 2020 by the American Thoracic Society. 10.1164/rccm.202003-0543OC PMC7258652 32242738

[30] Macmillan Types of head and neck cancer. Available online: http://www.macmillan.org.uk/informationand-support/head-and-neck-cancers/understanding-cancer/types-head-neck-cancer.html. Accessed 30 Sept 2018. Viruses. 2019;11(922):13–19.

[31] NCI Head and Neck Cancers. Available online: https://www.cancer.gov/types/head-and-neck/head-neckfact-sheet. Accessed 3 Dec 2017.

[32] Ndiaye C, Mena M, Alemany L (2014). HPV DNA, E6/E7 mRNA, and p16 INK4a detection in head and neck cancers: a systematic review and meta-analysis. Lancet Oncol.

[33] Franco P, Montagnani F, Arcadipane F, et al. The prognostic role of hemoglobin levels in patients undergoing concurrent chemo-radiation for anal cancer. Radiat Oncol. 2018, May;13(1):83. 10.1186/s13014-018-1035-9 PMC5930791 29720197

[34] De Marco F, Bucaj E, Foppoli C, et al. Oxidative stress in HPV-driven viral carcinogenesis: redox proteomics analysis of HPV-16 dysplastic and neoplastic tissues. PLoS One. 2012;7(3):e34366. 10.1371/journal.pone.0034366 PMC3314612 22470562

[35] Bodily JM, Mehta KPM, Cruz L (2011). The E7 open reading frame acts in cis and in trans to mediate differentiation dependent activities in the human papillomavirus type 16 life cycle. J Virol.

[36] Bodily JM, Mehta KPM, Laimins L (2011). Human papillomavirus E7 enhances hypoxia-inducible factor 1-mediated transcription by inhibiting binding of histone deacetylases. Cancer Res.

[37] Spriggs CC, Laimins LA, Spriggs C (2017). Human papillomavirus and the DNA damage response: exploiting host repair pathways for viral replication. Viruses.

[38] Songock WK, Kim SM, Bodily JM (2017). The human papillomavirus E7 oncoprotein as a regulator of transcription. Virus Res.

[39] Reiser J, Hurst J, Voges M, et al. High-risk human papillomaviruses repress constitutive kappa interferon transcription via E6 to prevent pathogen recognition receptor and antiviral-gene expression. J Virol. 2011;85:11372–11380. 10.1128/JVI.05279-11. 10.1128/JVI.05279-11 PMC3194958 21849431

[40] Chang YE, Laimins LA (2000). Microarray analysis identifies interferon inducible genes and Stat-1 as major transcriptional targets of human papillomavirus type 31. J Virol.

[41] Rincon-Orozco B, Halec G, Rosenberger S (2009). Epigenetic silencing of interferon-kappa in human papillomavirus type 16-positive cells. Cancer Res.

[42] Kaczkowski B, Morevati M, Rossing M (2016). A decade of global mRNA and miRNA profiling of HPV-positive cell lines and clinical specimens. Open Virol J.

[43] Kaczkowski B, Rossing M, Andersen DK (2012). Integrative analyses reveal novel strategies in HPV11,-16 and -45 early infection. Sci Rep.

[44] Karim R, Meyers C, Backendorf C (2011). Human papillomavirus deregulates the response of a cellular network comprising of chemotactic and proinflammatory genes. PLoS ONE.

[45] Merrick DT, Winberg G, McDougall JK (1996). Re-expression of interleukin 1 in human papillomavirus 18 immortalized keratinocytes inhibits their tumorigenicity in nude mice. Cell Growth Differ.

[46] Pahne-Zeppenfeld J, Schröer N, Walch-Rückheim B (2014). Cervical cancer cell-derived interleukin-6 impairs CCR7-dependent migration of MMP-9-expressing dendritic cells. Int J Cancer.

[47] Cheng YW, Lee H, Shiau MY (2008). Human papillomavirus type 16/18 upregulates the expression of interleukin-6 and antiapoptotic Mcl-1 in non-small cell lung cancer. Clin Cancer Res.

[48] Torres-Poveda K, Bahena-Román M, Madrid-González C (2014). Role of IL-10 and TGF-β1 in local immunosuppression in HPV-associated cervical neoplasia. World J Clin Oncol.

[49] N. Munoz, X. Castellsague, A.B. de Gonzalez, L. Gissmann, Chapter 1: HPV in the etiology of human cancer, Vaccine 24 (Suppl. 3). 2006. 10.1016/j.vaccine.2006.05.115 S3/1–10, S0264–410X(06)00591–3. 10.1016/j.vaccine.2006.05.115 16949995

[50] Pacini L, Savini C, Ghittoni R, et al. Downregulation of toll-like receptor 9 expression by beta human papillomavirus 38 and implications for cell cycle control. J Virol. 2015;89(22):11396-405. 10.1128/JVI.02151-15 (JVI.02151–15). 10.1128/JVI.02151-15 PMC4645680 26339055

[51] C. Martinez-Campos, A.I. Burguete-Garcia, V. Madrid-Marina. Role of TLR9 in oncogenic virus-produced cancer, Viral Immunol. 2017;30:98–105. 10.1089/vim.2016.010. 10.1089/vim.2016.0103 28151089

[52] Spencer LA, Weller PF (2010). Eosinophils and Th2 immunity: contemporary insights. Immunol Cell Biol.

[53] Aceves SS, Broide DH (2008). Airway fibrosis and angiogenesis due to eosinophil trafficking in chronic asthma. Curr Mol Med.

[54] Marks MA, Viscidi RP, Chang K (2011). Differences in the concentration and correlation of cervical immune markers among HPV positive and negative perimenopausal women. Cytokine.

[55] Wang L, Simons DL, Lu X (2019). Connecting blood and intratumoral T_reg_ cell activity in predicting future relapse in breast cancer. Nat Immunol.

[56] Zhang X, Li J, Peng Q, et al. Association of markers of systemic and local inflammation with prognosis of patients with rectal cancer who received neoadjuvant radiotherapy. Cancer Manag Re*s*. 2019;11:191–199 10.2147/CMAR.S187559. 10.2147/CMAR.S187559 PMC6307689 30636893

[57] Barker HE, Paget JT, Khan AA (2015). The tumour microenvironment after radiotherapy: mechanisms of resistance and recurrence. Nat Rev Cancer.

[58] Gameiro SR, Jammeh ML, Wattenberg MM (2014). Radiation-induced immunogenic modulation of tumor enhances antigen processing and calreticulin exposure, resulting in enhanced T-cell killing. Oncotarget.

[59] Andersson U, Wang H, Palmblad K (2000). High mobility group 1 protein (HMG-1) stimulates proinflammatory cytokine synthesis in human monocytes. J Exp Med.

[60] Lotfi R, Herzog GI, DeMarco RA (2009). Eosinophils oxidize damage-associated molecular pattern molecules derived from stressed cells. J Immunol.

[61] Lajer CB, Von Buchwald C (2010). The role of human papillomavirus in head and neck cancer. APMIS.

[62] Liu C, Mann D, Sinha UK (2018). The molecular mechanisms of increased radiosensitivity of HPV-positive oropharyngeal squamous cell carcinoma (OPSCC): an extensive review. J Otolaryngol Head Neck Surg.

[63] Kimple RJ, Smith MA, Blitzer GC (2013). Enhanced radiation sensitivity in HPVpositive head and neck cancer. Cancer Res.

[64] Ziemann F, Arenz A, Preising S (2015). Increased sensitivity of HPV-positive head and neck cancer cell lines to x-irradiation ± cisplatin due to decreased expression of E6 and E7 oncoproteins and enhanced apoptosis. Am J Cancer Res.

[65] Rieckmann T, Tribius S, Grob TJ (2018). HNSCC cell lines positive for HPV and p16 possess higher cellular radiosensitivity due to an impaired DSB repair capacity. Radiother Oncol.

[66] Lassen P, Eriksen JG, Hamilton-Dutoit S (2018). HPV-associated p16-expression and response to hypoxic modification of radiotherapy in head and neck cancer. Radiother Oncol.

[67] Barker HE, Paget JTE, Khan A, Harrington KJ (2015). The tumour microenvironment after radiotherapy: mechanisms of resistance and recurrence. Nat Rev Cancer.

[68] Mirghani H, Amen F, Tao Y (2015). Increased radiosensitivity of HPV-positive head and neck cancers: molecular basis and therapeutic perspectives. Cancer Treat Rev.

[69] Zhu J, Wang H, Gao MJ (2019). Prognostic values of lymphocyte and eosinophil counts in resectable cervical squamous cell carcinoma. Future Oncol.

