# Supplementary figures and images for: The Prognostic Role of Baseline Eosinophils in HPV-Related Cancers: a Multi-institutional Analysis of Anal SCC and OPC Patients Treated with Radical CT-RT
Source: J Gastrointest Cancer. 2022 Aug 1;54(2):662–71. doi: 10.1007/s12029-022-00850-y (PMC9342937; doi:10.1007/s12029-022-00850-y)

## Slide 1
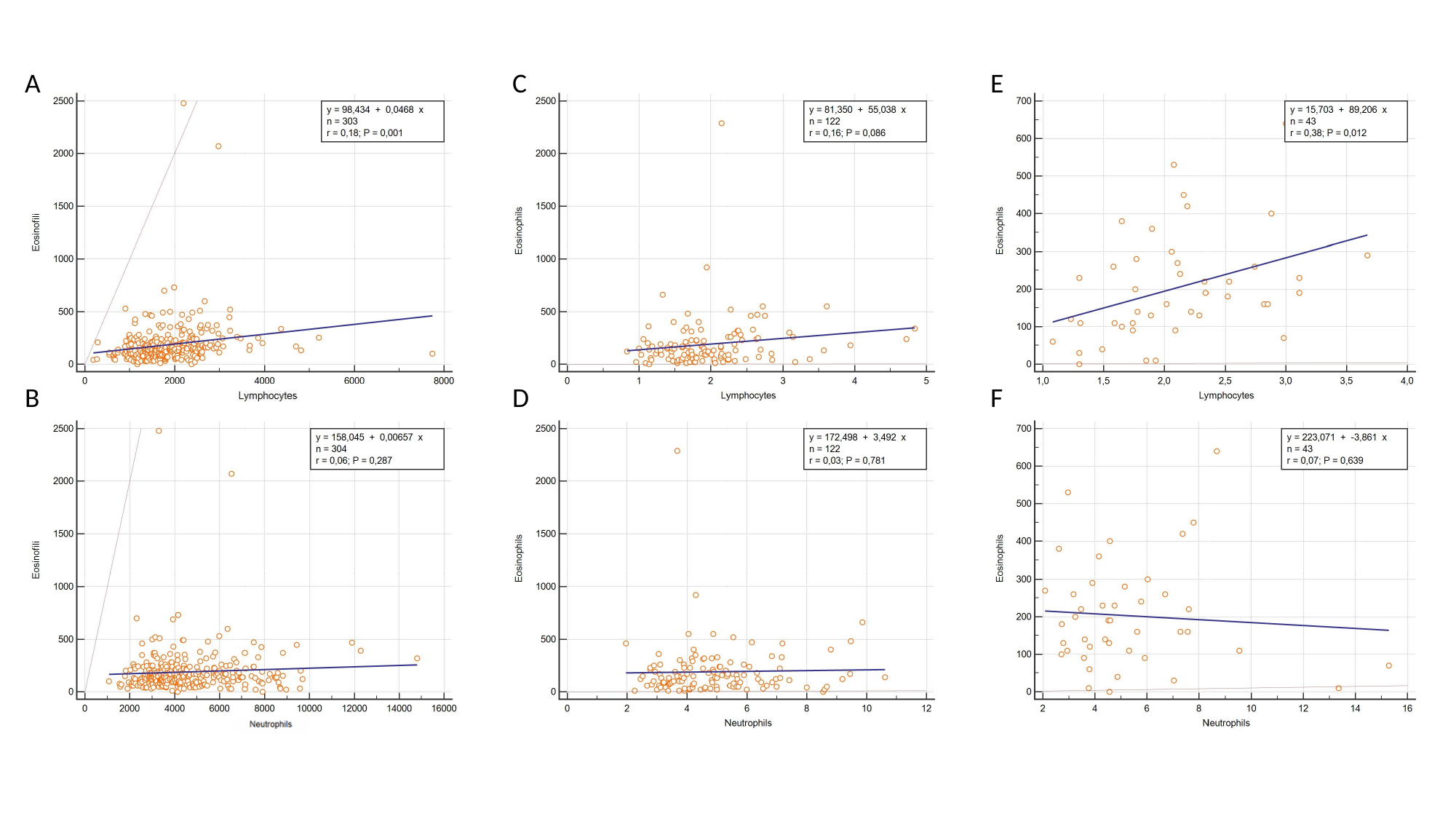

A
C
E
B
D
F

Supplement: Supplementary file 1 — Supplementary file1 (PPTX 1.52 MB) [file 12029_2022_850_MOESM1_ESM.pptx]
